# Transcutaneous spinal cord stimulation combined with robotic-assisted body weight-supported treadmill training enhances motor score and gait recovery in incomplete spinal cord injury: a double-blind randomized controlled clinical trial

**DOI:** 10.1186/s12984-025-01545-8

**Published:** 2025-01-30

**Authors:** Natalia Comino-Suárez, Juan C. Moreno, Álvaro Megía-García, Antonio J. del-Ama, Diego Serrano-Muñoz, Juan Avendaño-Coy, Ángel Gil-Agudo, Mónica Alcobendas-Maestro, Esther López-López, Julio Gómez-Soriano

**Affiliations:** 1 Toledo Physiotherapy Research Group (GIFTO), Faculty of Physiotherapy and Nursing of Toledo, Universidad de Castilla-La Mancha, Toledo, Spain; 2 BioRobotics Group, Center for Automation and Robotics, CSIC-UPM, Spanish National Research Council, Ctra. Campo Real Km 0,2., 28500 Arganda del Rey, Madrid, Spain; 3 Unit of Neurorehabilitation, Biomechanics and Sensorimotor Function (HNP-SESCAM), Associated Unit of R&D&I to the CSIC, Toledo, Spain; 4 Bioengineering Systems and Technologies Research Group, School of Science and Technology, Rey Juan Carlos University, Móstoles, Madrid Spain; 5 Biomechanics and Technical Aids Unit, National Hospital for Paraplegics, Toledo, Spain; 6 Department of Physical Medicine and Rehabilitation, National Hospital for Paraplegics, Toledo, Spain; 7 Department of Physical Therapy, National Hospital for Paraplegics, Toledo, Spain; 8 Department of Physical Therapy, Faculty of Health Sciences, Universidad Alfonso X El Sabio, Madrid, Spain

**Keywords:** Spinal cord injury, Transcutaneous spinal cord stimulation, Lokomat, Robotic-assisted gait training, Motor function, Gait rehabilitation

## Abstract

**Background:**

Although transcutaneous spinal cord stimulation (tSCS) has been suggested as a safe and feasible intervention for gait rehabilitation, no studies have determined its effectiveness compared to sham stimulation.

**Objective:**

To determine the effectiveness of tSCS combined with robotic-assisted gait training (RAGT) on lower limb muscle strength and walking function in incomplete spinal cord injury (iSCI) participants.

**Methods:**

A randomized, double-blind, sham-controlled clinical trial was conducted. Twenty-seven subacute iSCI participants were randomly allocated to tSCS or sham-tSCS group. All subjects conducted a standard Lokomat walking training program of 40 sessions (5 familiarization sessions, followed by 20 sessions combined with active or sham tSCS, and finally the last 15 sessions with standard Lokomat). Primary outcomes were the lower extremity motor score (LEMS) and dynamometry. Secondary outcomes included the 10-Meter Walk Test (10MWT), the Timed Up and Go test (TUG), the 6-Minute Walk test (6MWT), the Spinal Cord Independence Measure III (SCIM III) and the Walking Index for Spinal Cord Injury II (WISCI-II). Motor evoked potential (MEP) induced by transcranial magnetic stimulation (TMS) were also assessed for lower limb muscles. Assessments were performed before and after tSCS intervention and after 3-weeks follow-up.

**Results:**

Although no significant differences between groups were detected after the intervention, the tSCS group showed greater effects than the sham-tSCS group for LEMS (3.4 points; p = 0.033), 10MWT (37.5 s; p = 0.030), TUG (47.7 s; p = 0.009), and WISCI-II (3.4 points; p = 0.023) at the 1-month follow-up compared to baseline. Furthermore, the percentage of subjects who were able to walk 10 m at the follow-up was greater in the tSCS group (85.7%) compared to the sham group (43.1%; p = 0.029). Finally, a significant difference (p = 0.049) was observed in the comparison of the effects in the amplitude of the rectus femoris MEPs of tSCS group (− 0.97 mV) and the sham group (− 3.39 mV) at follow-up.

**Conclusions:**

The outcomes of this study suggest that the combination of standard Lokomat training with tSCS for 20 sessions was effective for LEMS and gait recovery in subacute iSCI participants after 1 month of follow-up.

*Trial registration* ClinicalTrials.gov (NCT05210166).

**Supplementary Information:**

The online version contains supplementary material available at 10.1186/s12984-025-01545-8.

## Introduction

Spinal cord injury (SCI) is a devastating condition with a significant impact on a person’s life [1]. According to recent estimates from the National Spinal Cord Injury Statistical Center, nearly 70% of SCI cases are incomplete (iSCI) [2]. The restoration and improvement of walking ability is a high priority after injury and is a common target in the rehabilitation process [3].

Robot-assisted gait training (RAGT) is a form of intensive locomotor training that enables early initiation of gait training in severely dependent patients [4]. Although the efficacy of RAGT has been demonstrated, there is no clear superiority to other gait training approaches, and the benefits appear to be modest [4–6]. In recent years, there has been growing interest in combining neuromodulation strategies with gait training interventions with promising results [7–9].

Transcutaneous spinal cord stimulation (tSCS) is a non-invasive neuromodulation strategy in which electrodes are applied to the skin over vertebrae and used to stimulate spinal circuits via an electrical current [10–12]. At the spinal level, tSCS has shown the ability to activate afferent nerve fibers within posterior spinal roots, akin to the neural structures stimulated with epidural electrical stimulation [11, 13]. Consequently, tSCS has emerged as a promising and clinically valuable adjunct to physical therapy interventions, avoiding the risks associated with surgical procedures.

Previous studies have combined transcutaneous stimulation of the lumbar segments of the spinal cord with several therapeutic interventions, such as treadmill stepping [14–16], mobilization [17] postural control exercises [18], RAGT [19, 20], or portable exoskeletons [21, 22] to improve motor outcomes in individuals with iSCI. The safety and feasibility of the combination of tSCS with locomotor training have been evidenced [10] and this combination has been associated with improved walking [15, 17, 20, 21, 23, 24] increased volitional muscle activation [14, 15, 23] and decreased spasticity [25]. However, the real effectiveness of tSCS has not been yet established due to strong limitations in previous studies, such as reduced sample size or lack of a control group [10, 26].

Corticospinal excitability plays a critical role in the motor recovery and functional rehabilitation of the sub-acute SCI [27]. Corticospinal integrity can improve following locomotor function and may potentially be enhanced with neuromodulation techniques [28]. Although transcutaneous direct current stimulation has evidenced an increase of corticospinal excitability [29], the effect of pulsed tSCS on motor evoked potentials (MEPs) are still unknown. Furthermore, no previous studies have evaluated the success of the blinding of participants and assessors to adequately validate the sham stimulation protocol. Therefore, although tSCS is a low burden and safe therapy with encouraging results, there is a need for high-quality controlled studies to determine the full potential and effectiveness of tSCS in iSCI patients.

Most of the previous studies in the field of tSCS recruited subjects at the chronic stage of the SCI [10, 26]. However, the vast majority of recovery occurs in the first 3 months, although a small amount can persist for up to 18 months or longer [30]. The sub-acute phase of SCI has a higher implication of neuroplastic mechanisms that are limited during spontaneous recovery but can be enhanced by neuromodulation strategies [28]. Very few studies have been published in subjects during the sub-acute phase of the SCI stimulating at the lumbosacral [20] or cervical [31] levels. Due to this, it is crucial to conduct trials during the sub-acute phase of SCI to leverage the heightened plasticity of the nervous system currently.

The main aim of this randomized, double-blind, sham-controlled, clinical trial was to investigate the effectiveness of a program of 20 sessions of lumbosacral tSCS combined with RAGT on the strength of the lower limbs of sub-acute iSCI participants compared to sham tSCS and RAGT. The secondary objectives were to evaluate the effect of tSCS on gait recovery, independence, hypertonia and corticospinal activity. Finally, to validate the sham stimulation protocol for future studies, the success of the blinding of participants and assessor was also quantified.

## Methods

### Study design

This randomized, double-blind (participants and assessor) sham-controlled clinical trial was conducted at the National Hospital for Paraplegics (Toledo, Spain). Prior approval for the study was obtained from the local ethics committee, and the protocol was registered at ClinicalTrials.gov (NCT05210166). The trial followed the principles of the Declaration of Helsinki [32] and the standard protocol of the CONSORT Statement [33].

### Participants

Participants who met the following inclusion criteria were enrolled: (1) spinal cord injury at level C2-T11, classified as grades C-D in the American Spinal Injury Association Impairment Scale (AIS); (2) age older than 18 years; (3) less than 6 months from the injury; and (4) grade of hypertonia and frequency of spasms less than 3 on the Modified Ashworth Scale [34] and Penn Spam Frequency Scale, respectively [35]. Individuals were excluded if they met any of the following criteria: (1) metal implant in the stimulation area; (2) implanted electronic devices; (3) history of epilepsy; (4) inability to properly adjust the harness or straps (colostomy bags, uncorrectable differences in leg length or skin lesions); (5) fixed joint contractures that limit the range of motion; (6) considerably reduced bone density (osteopenia or osteoporosis); (7) body weight more than 135 kg or taller than 1.95 m; (8) non-consolidated fractures; (9) pregnancy; or (10) tumour process.

### Randomization and blinding

A researcher performed the randomization using the web-based tool “Randomization Plans” (http://www.jerrydallal.com/random/random_block_size_r.htm). Participants were randomly assigned to one of two groups: tSCS or sham tSCS. The allocation was concealed until participants were enrolled and assigned to the intervention group. All team members were blinded, except for the therapist that applied the spinal cord stimulation.

### Intervention

**Fig. 1:**
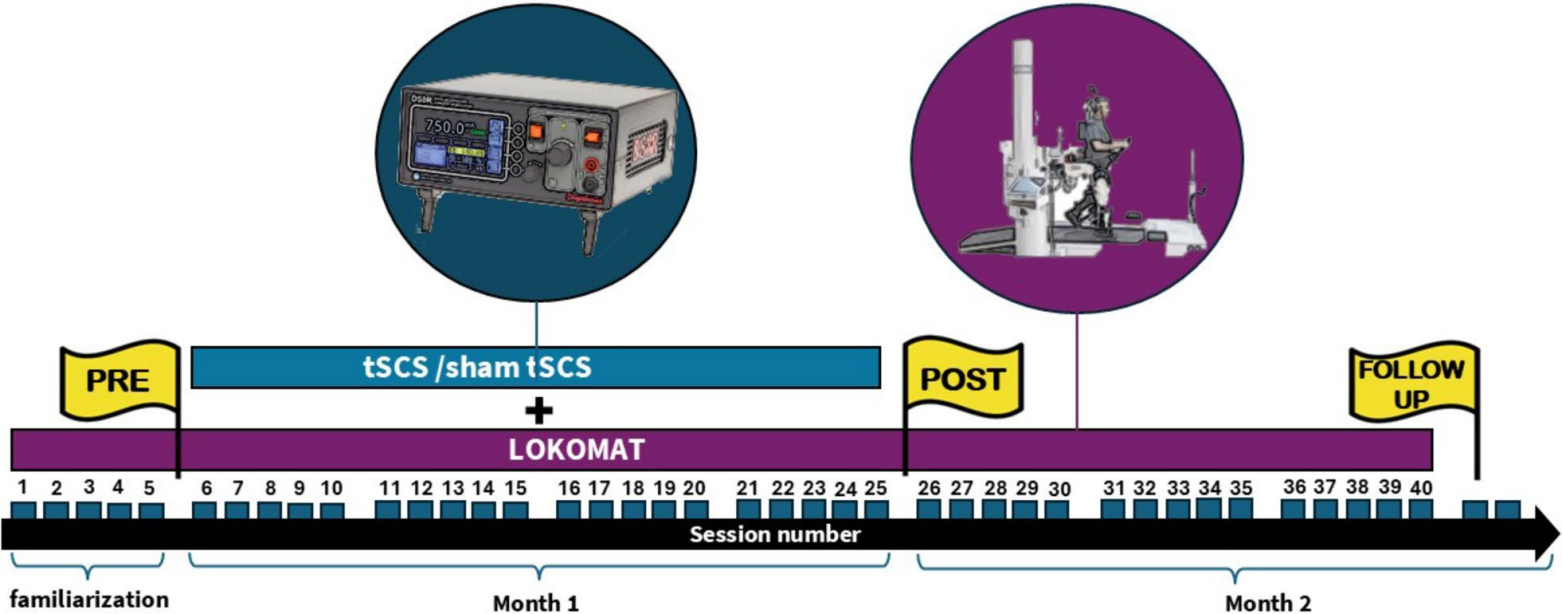
Scheme of the design of the study. Yellow flags represent assessments before the intervention (PRE), after the 20 sessions of active/sham transcutaneous spinal cord stimulation (POST) and after finishing the Lokomat protocol (FOLLOW-UP)

All the participants completed a total of 40 walking training sessions on the Lokomat (Hocoma, Switzerland) over 8 weeks (5 sessions/week), with each session lasting 30 min. The first 5 sessions involved familiarization with the Lokomat alone. For the next 20 sessions, tSCS combined with the Lokomat was applied. The remaining 15 sessions continued with Lokomat alone (Fig. 1). This design was chosen to not interfere with the hospital-based Lokomat walking training clinical protocol. At each session, after the first 5 min of gait training with the Lokomat alone, tSCS or sham tSCS was added for 20 min. The amount of body weight support was initially set at 60% of each individual’s weight and was adjusted as needed based on load tolerance, ensuring a minimum support level of 25%. The walking speed and level of robotic assistance were tailored to the participants’ characteristics according to the clinical criteria adopted by the protocol of the rehabilitation unit.

#### Transcutaneous spinal cord stimulation (tSCS)

Electrical stimulation was applied to all subjects using a constant current stimulator (DS8R, Digitimer Ltd., UK) in open loop system (without communication with the Lokomat), which delivered a symmetrical rectangular biphasic current at a frequency of 30 Hz and a 1 ms pulse-width, with a phase duration of 0.5 ms and inter-pulse-interval of 0. Three self-adhesive pre-gelled flexible carbon surface electrodes (9 × 5 cm, ValuTrode, Axelgaard Manufacturing Co., USA) were used. The anode was placed on the thoracic spinal cord along the midline between the spinous processes T11–T12, and two interconnected cathodes were placed symmetrically on the abdomen on both sides of the umbilicus. This configuration ensured a higher current density at the spinal level and has been used in previous studies [36]. The stimulation intensity was set at the patient’s tolerance threshold in each session. At this intensity, paresthesias in the lower limbs were commonly reported by patients. Whenever stimulation evoked discomfort, the therapist decreased the stimulation intensity to a tolerable level. If the subject habituated to the current during the session, the intensity was increased until the participant achieved the same intense but tolerable sensation.

#### Sham transcutaneous spinal cord stimulation (sham-tSCS)

The same stimulation protocol with the same device used for the experimental group was used, with the only difference being the stimulus intensity, which was set at the sensory threshold, maintained for 30 s, and gradually decreased to zero for the remaining 20 min of the intervention. Participants received the same instructions as those given during the active tSCS session. This method has previously been used in a tSCS study in non-injured subjects [36].

### Outcome measures

Outcomes were measured at three time points: pre-intervention, after the 20 sessions of tSCS (post), and between 1 and 3 days after finishing the Lokomat intervention (follow-up). This range was included to meet subjects and/or assessor availability within the clinical context. Sociodemographic and clinical data of the participants were collected at baseline. Adverse events were systematically registered throughout the intervention.

The main outcome variable was lower extremity motor strength and was measured using the Lower Extremity Motor Score (LEMS) and dynamometry. LEMS subscale (0–50) is derived from the International Standards for Neurological Classification of Spinal Cord Injury (ISNCSCI) [37] and was assessed by two physicians certified by the American Spinal Injury Association (ASIA). All other outcomes were assessed by the same assessor. Strength was measured as the isometric maximal voluntary contraction (MVC) of knee extensors and ankle dorsiflexors of both limbs using the hand-held dynamometer Micro Fet 2TM (Hoggan Scientific, LLC, USA). The MVC was determined with the participant seated, with knee and hip flexed at 90° to evaluate knee extensors and in supine position for ankle dorsiflexor muscles. Participants were instructed to perform contractions for 3–5 s with 1-min breaks between contractions [36]. The dynamometry data were recorded as the average of three repetitions of each contraction.

As secondary outcomes, walking, hypertonia, independence and motor-evoked potential assessments were conducted. Walking speed was evaluated using the 10-m walk test (10MWT) [38]. The 6-min walk test (6MWT) measures the distance (in meters) covered within 6 min to assess endurance. The Timed Up and Go test (TUG) [38] was used to evaluate dynamic balance and gait speed. Walking capacity was defined as the ability to complete the 10-m walk test (10MWT) and was registered as a dichotomous outcome (able/unable to walk 10 m). By including this outcome, we can measure and analyze the recovery of gait in subjects who cannot perform gait tests (10MWT, 6MWT or TUG) in the early stages.

Muscle resistance to passive movement was evaluated using the Modified Ashworth Scale (MAS) (0–4) [34] for both lower limbs. The level of independence in activities of daily living was assessed using the Spinal Cord Independence Measure III (SCIM III) [39]. This scale comprises 19 items and higher scores indicate a greater degree of independence. Additionally, the Walking Index for Spinal Cord Injury II scale (WISCI-II) was used to score patients on a scale from 0 to 20, considering the technical aids, orthoses, and assistance required for walking 10 m [40].

Motor evoked potentials (MEP) of the rectus femoris (RF) and tibialis anterior (TA) muscles were elicited by transcranial magnetic stimulation (TMS) using a Magstim Rapid 2 device (Magstim Company Ltd., UK) equipped with a double-cone coil. The optimal stimulation site (hot spot, the area where TMS elicited the largest MEP) was identified for both the RF and TA muscles individually. The electromyographic signals were recorded using bipolar silver chloride electrodes (×1000 amplification) filtered with a 20–450 Hz bandpass filter (Signal Conditioning Electrodes v2.3, Delsys Inc., USA). Electrodes were placed over the RF of the belly of the quadriceps muscle and the proximal third region of the TA following the SENIAM recommendations. The active motor threshold was defined as the lowest stimulus intensity that induced a MEP with an amplitude of at least 100 μVs in at least 3 of a series of 5 stimuli during slight tonic contraction of the target muscle (approximately 20% of the isometric MVC). Test MEPs were recorded in supine position during slight tonic contraction of the target muscle (approximately 20% of the isometric MVC) as an average of 10 single-pulse stimuli applied at 110% of the motor threshold. The average MEP peak-to-peak amplitude and latency were analysed manually for each subject using the analysis software Signal v.5 (Cambridge Electronic Design, UK). This protocol has shown high reliability in a previous study in healthy volunteers [36].

After finalizing the intervention, the blinding success of the participants and the assessor were evaluated through a questionnaire with five closed questions following a protocol validated in previous studies [36, 41]. (1) “Strongly believe the applied intervention is new treatment”; (2) “Somewhat believe the applied intervention is new treatment”; (3) “Somewhat believe the applied intervention is a placebo”, (4) “Strongly believe the applied intervention is a placebo”, or (5) “Do not Know” [41].

### Data recording and statistical analysis

The analysis of clinical outcomes was performed on an intention-to-treat basis, with missing data for participants lost during follow-up imputed using the values from their last observation carried forward. This imputation method is the simplest imputation method and is usually recommended in clinical studies [42]. All the data are expressed as the mean and standard deviation. For the 10MWT and TUG, the worst value of all participants was assigned to participants who were unable to perform the gait tests. This type of missing data is called “Missing Not at Random”, and the “worst value” is considered a conservative method of imputation data [43]. The analysis of MEP was not performed on an intention-to-treat basis due to external problems with the TMS device and the elevated missing data for this outcome. Only data of recorded MEP were included in the statistical analysis. The effect of each outcome was calculated as the change from baseline of the scores at post- and follow-up within each group.

The statistical analysis was conducted using SPSS 28.0 software (SPSS Inc., USA). A descriptive analysis of socio-demographic variables was performed. Normal distribution and homogeneity were examined using the Shapiro‒Wilk test and Levene test, respectively, to determine a parametric or nonparametric analysis. Comparisons of socio-demographic and background clinical characteristics between groups were conducted using independent sample t-tests for quantitative data and the chi-square test for categorical data. Intra-group changes were analysed with one-way repeated-measures ANOVA when parametric conditions were met. If the sphericity assumption was violated (Mauchly’s test: p < 0.05), the Greenhouse‒Geisser correction was applied. The Bonferroni test was used for post hoc comparisons. In the case of non-parametric conditions, the Friedman test and post hoc Wilcoxon t-test were applied. Between-group differences were analysed by comparing the effect from baseline of both groups using the independent samples t-test when parametric conditions were met and the Mann‒Whitney-U test when non-parametric conditions were assumed. Categorical variables were compared between groups through Pearson’s Chi^2^ and Fisher’s Exact test for frequencies < 5. Statistical significance was set at p < 0.05 for all variables.

The blinding analysis was performed using Stata Version 15.1 software (StataCorp., College Station, TX, USA). Successful blinding was determined using the James and Bang indexes [41, 44]. James’ BI infers overall success of blinding in all arms. It ranges from 0 to 1 (0 = total lack of blinding, 1 = complete blinding, 0.5 = completely random blinding). Values of < 0.5 for the upper limit of the confidence interval were considered indicative of no blinding [44]. The Bang’s index assesses the successful blinding of each study arm. This index ranges from 0 to 1, being “0” a total lack of blinding, “1” being complete blinding and “0.5” being completely random blinding (i.e. 50% correct and 50% incorrect guesses). If the upper bound of the confidence interval of the Bang Index is below 0.5 (i.e. confidence interval does not cover the null value), the study is regarded as lacking blinding [41].

## Results

### Baseline socio-demographic and clinical characteristics of participants

**Fig. 2:**
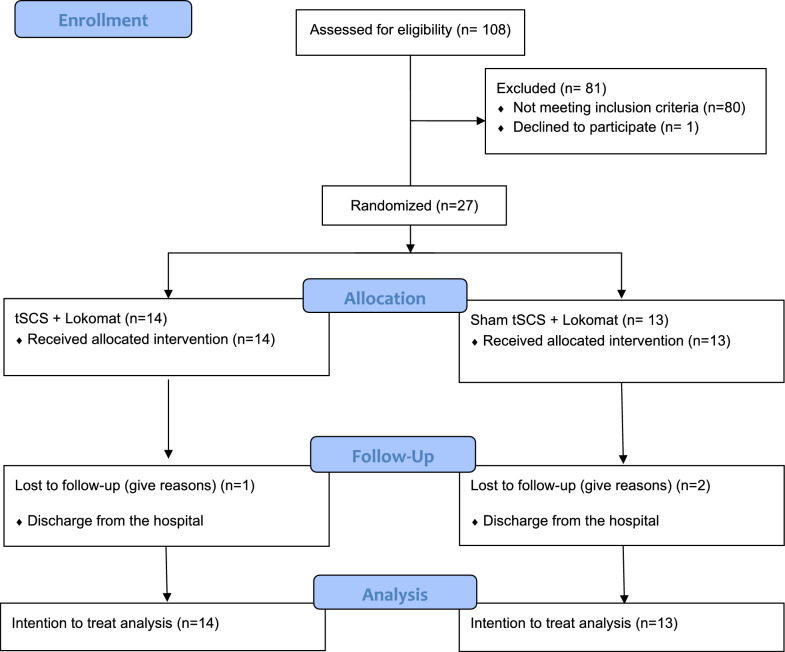
CONSORT flow diagram of the participants

Participants affected by iSCI in the sub-acute phase were assessed for eligibility to participate in this study (n = 108). Twenty-seven participants met the inclusion criteria, signed the consent form, and were randomly allocated into two groups: tSCS (n = 14) and sham-tSCS (n = 13) (Fig. 2). No differences between the groups were found at baseline for age, time since SCI, AIS or aetiology. An almost significant difference was found for sex, weight, height and body mass index. Regarding the percentage of body weight support (BWS) of the Lokomat sessions, there was a higher discharge in the active group compared to the sham group in the first session (p = 0.035) and in the 20th session (p = 0.063) (see Table 1). However, the overall reduction in BWS during the intervention was comparable between the groups, with a decrease of 7.97% in the active group and 6.92% in the sham group. Notably, both groups showed a reduction in BWS over the course of the treatment. Specific data for each subject are reported in Additional file 1. The average tSCS intensity applied in the active tSCS group was 50.9 mA (SD 9.3).

**Table 1:** Sociodemographic and clinical characteristics of SCI patients

	Total sample (n = 27)	tSCS + Lokomat (n = 14)	Sham tSCS + Lokomat (n = 13)	Intergroups differences (p value)
Age (years), mean (SD)	48 (15.36)	47.36 (14.45)	48.69 (16.84)	*p* = 0.82^a^
Male, n (%)	24 (88.9%)	14 (100%)	10 (76.9%)	*p* = 0.057^b^
Weight (Kg), mean (SD)	76.67 (17.1)	82.64 (15.26)	70.23 (17.11)	*p* = 0.057^a^
Height (m), mean (SD)	1.74 (0.10)	1.75 (0.07)	1.72 (0.13)	*p* = 0.37^a^
BMI (kg/m^2^), mean (SD)	25.18 (4.31)	26.71 (4.11)	23.53 (4.05)	*p* = 0.054^a^
Time since SCI (days), mean (SD)	111.33 (40.49)	109.14 (35.3)	113.69 (46.8)	*p* = 0.777^a^
% BWS Lokomat, mean (SD)				
1st session	37.96% (9.02)	41.43% (9.29)	34.23% (7.31)	*p* = 0.035^a^
20th session	30.38% (8.47%)	33.46% (10.48)	27.31% (4.38)	*p* = 0.063^a^
AIS, n (%)				
C	18 (66.7%)	8 (57.1%)	10 (76.9%)	*p* = 0.276^b^
D	9 (33.3%)	6 (42.9%)	3 (23.1)	
Cause of injury n (%)				
Traumatic	15 (55.6%)	7 (50%)	8 (61.5%)	p = 0.258^b^
Inflammatory	3 (11.1%)	3 (21.4%)	–	
Vascular	6 (22.2%)	2 (14.3%)	4 (30.8%)	
Post-surgical	3 (11.1%)	3 (14.3%)	1 (7.7%)	

*BMI* Body mass Index, *BWS* % Body weight support, *AIS* American Spinal Cord Injury Association Impairment Scale. Statistical test: a) Student’s t-test independent samples. b) Pearson’s chi-squared test

### Effects on lower extremity motor strength and hypertonia

Table 2 shows the values obtained for the main outcome variables and hypertonia. No statistically significant differences were found between groups at baseline. The total Lower Extremity Motor Score (LEMS) showed a statistically significant increase in both groups at post-intervention and at follow-up compared with baseline (*p* < 0.05). The active tSCS group showed significantly greater improvement than the sham group at follow-up (*p* = 0.033). In the active tSCS group, the knee extensor maximal voluntary contraction (MVC) increased at post-treatment (6.19 kgf, *p* = 0.016) and follow-up (10.22 kgf, *p* = 0.002), whereas the MVC in the sham group increased only at post-treatment (6.20 kgf, *p* = 0.027) compared to baseline. For MVC of ankle dorsiflexors, a significant increase was observed at follow-up from baseline in the tSCS group (4.9 kgf, *p* = 0.003) that it was not detected in the sham group (3.4 kgf, *p* = 0.054). However, the effect on MVC of both knee extensors and ankle dorsiflexors was not significantly different between groups. Regarding hypertonia, no statistically significant changes were found in the Modified Ashworth score (MAS) scores of the lower extremities in any of the comparisons.

**Table 2:** Values are represented as mean and standard deviation (SD)

Outcomes	Assessment	Intervention group (n = 14) (tSCS + Lokomat)		Control group (n = 13) (sham tSCS + Lokomat)		Comparison of changes Intervention vs. control Mean difference (95% CI)	
		Mean (SD)	Change score	Mean (SD)	Change score	Post	Follow-up
LEMS total score	Pre-	25.36 (11.81)	4.14 (3.21)* ***p* < 0.001**	27.31 (14.47)	2.62 (3.38)* ***p* < 0.05**	1.52 (1.08 to 4.14)^⌘^	**3.37** **(0.29 to 6.46)**^⌘^ ***p* < 0.05**
	Post-	29.50 (12.01)		29.92 (15.32)			
	Follow-up	32.50 (12.63)	7.14 (4.07)* ***p* < 0.001**	31.08 (15.06)	3.77 (3.68)* ***p* < 0.01**		
MVC Knee extensors (Kgf)	Pre-	19.10 (11.53)	6.19 (7.79)* ***p* < 0.05**	15.27 (11.28)	6.20 (7.24)* ***p* < 0.05**	0.012 (− 6.11 to 6.14)^⌘^	3.62 (− 11.39 to 4.13)^⌘^
	Post-	25.29 (14.03)		21.47 (15.47)			
	Follow-up	29.32 (14.64)	10.22 (11.03)* ***p* < 0.01**	21.87 (16.53)	6.59 (7.44)*		
MVC Ankle dorsiflexors (Kgf)	Pre-	9.09 (7.64)	2.65 (3.36)*	7.14 (11.17)	1.23 (4.88)*	− 1.42 (− 4.87 to 2.01)^⌘^	− 1.48 (− 5.30 to 2.33)^⌘^
	Post-	11.75 (7.89)		8.38 (10.43)			
	Follow-up	13.96 (8.9)	4.87 (4.54)* ***p* < 0.001**	10.53 (13.71)	3.39 (4.68)*		
MAS score	Pre-	0.96 (0.98)	− 0.18 (0.69)^$^	1.04 (0.96)	− 0.11 (0.5)^$^	− 0.063 (− 0.54 to 0.42)^&^	0.19 (− 0.33 to 0.73)^&^
	Post-	0.78 (0.75)		0.92 (0.93)			
	Follow-up	0.89 (0.98)	− 0.07 (0.58)^$^	0.76 (0.80)	− 0.27 (0.75)^$^		

*LEMS* Lower extremity motor score, *MVC* Maximal voluntary contraction, *MAS* Modified Ashworth scale, *CI* Confidence interval. One-way repeated ANOVA and post-hoc Bonferroni (*); Friedmann test and Wilcoxon post-hoc (^$^). Intergroup comparison changes scores with t-student independent samples (^⌘^) and Mann–Whitney-U test (^&^). Bold font indicates statistical significance (*p* < 0.05)

### Functional outcomes

**Fig. 3:**
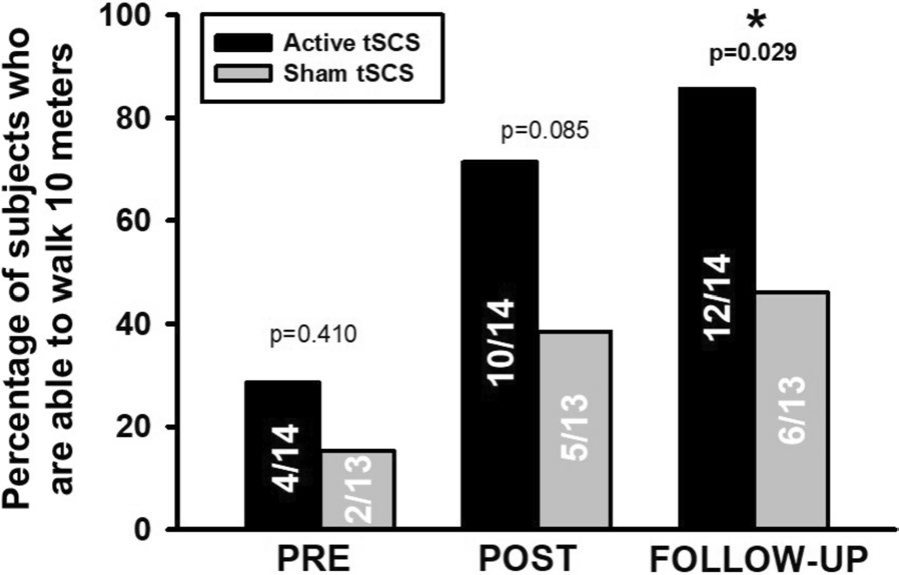
Proportion of subjects who were able to walk 10 m at each group for each assessment. The number of subjects is represented inside the bars. *: p < 0.05

**Fig. 4:**
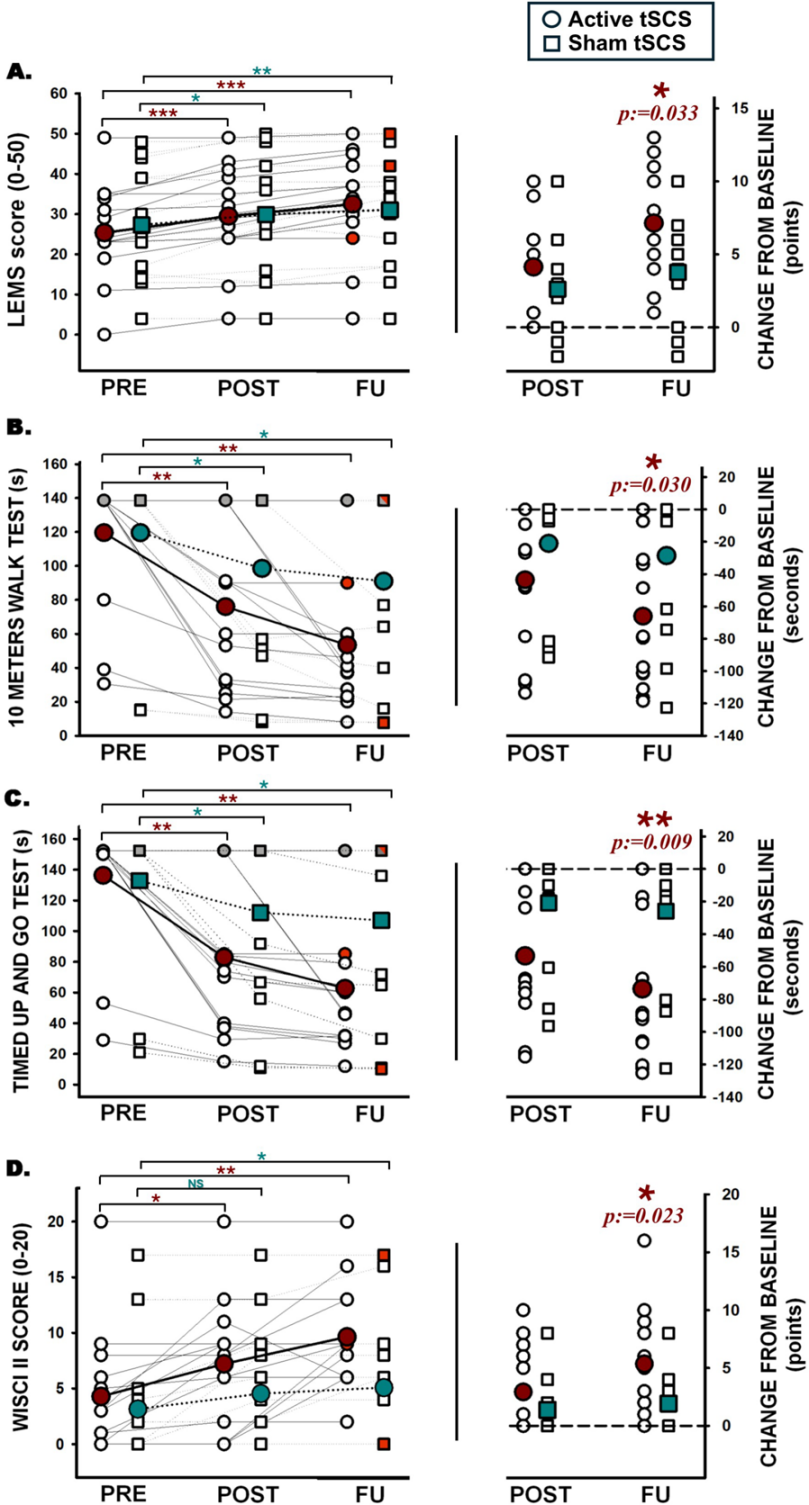
Individualized data for LEMS score (**A**), 10-m walking test (**B**), Timed Up and Go test (**C**) and Walking Index for Spinal Cord Injury II scale (**D**). Left panels show raw data at baseline (PRE), after the intervention (POST) and 1-month follow-up (FU). Right panels show the change from baseline at POST and at FU. Horizontal line of the right panels represents the “zero effect”. Brown circles represent the mean of the active transcutaneous spinal cord stimulation (tSCS) group and cyan squares represent the mean of the sham tSCS group. Filled in red represent the imputed value of the missing data of subjects lost during follow-up. Filled in grey represent the imputed data of subjects who were not able to finish the test. (s): seconds; *: p < 0.05; **: p < 0.01

Figure 3 illustrates the proportion of participants who were able to walk in each group for each assessment. Table 3 shows the values obtained for the functional outcomes. The proportion of participants who were unable to walk 10 m reported in Fig. 3, also were unable to complete the test of 10MWT, TUG and 6MWT and these data were imputed. tSCS showed a statistically significant improvement at post- and follow-up for all outcomes compared to baseline. Sham-tSCS also showed a significant improvement except for the Walking Index for Spinal Cord Injury II (WISCI-II) score at post-intervention. Regarding the comparison of the effects between groups, although no differences were detected after the intervention for any of the outcomes, the 10-m walk test (10MWT) (Mann‒Whitney test; *p* = 0.083) and The Timed Up and Go test TUG (Mann‒Whitney test; *p* = 0.054) showed almost significant differences. At the follow-up, significant differences were observed for the WISCI-II score (Mann‒Whitney test;* p* = 0.023), the 10MWT (Mann‒Whitney test; *p* = 0.030) and the TUG test (Mann‒Whitney test; *p* = 0.009). Individualized results for outcomes with statistical significance are reported in Fig. 4.

**Table 3:** Values represented as mean and standard deviation (SD)

Outcomes		Assessment	Intervention group (n = 14) (tSCS + Lokomat)		Control Group (n = 13) (sham tSCS + Lokomat)		Comparison of changes Intervention vs. control Mean difference (95% CI)	
			Mean (SD)	Change score	Mean (SD)	Change score	Post	Follow-up
10 MWT (seconds)		Pre-	119.58 (39.18)	− **43.57 (42.54)**^$^ **p < 0.01**	119.61 (46.34)	− **21.04 (37.69)**^$^ **p < 0.05**	− 22.52 (− 54.48 to 9.43)^&^	− **37.51 (**− **72.78 to **− **2.23)**^&^ **p < 0.05**
		Post-	76.01 (48.37)		98.56 (54.46)			
		Follow-up	53.46 (41.51)	− **66.12 (44.63)**^$^ **p < 0.01**	91.00 (56.90)	− **28.61 (44.39)**^$^ **p < 0.05**		
TUG (seconds)		Pre-	136.32 (40.62)	− **53.33 (45.58)**^$^ **p < 0.01**	132.86 (47.72)	− **20.81 (35.48)**^$^ **p < 0.05**	− 35.52 (− 65.08 to 0.04)^&^	− **47.70 (**− **82.52 to **− **12.87)**^&^ **p < 0.01**
		Post-	83.0 (50.40)		112.04 (56.90)			
		Follow-up	62.74 (43.19)	− **73.58 (45.58)**^$^ **p < 0.01**	106.98 (59.72)	− **25.88 (41.99)**^$^ **p < 0.05**		
6MWT (meters)	Pre-		18.41 (40.24)	**39.34 (39.86)**^$^	37.00 (90.56)	**43.06 (72.15)**^$^ **p < 0.01**	− 3.72 (− 49.29 to 42.01)^&^	12.75 (− 46.29 to 71.80)^&^
	Post-		57.75 (65.78)		80.06 (159.80)			
	Follow-up		87.42 (82.01)	**69.01 (61.01)**^$^ **p < 0.01**	93.26 (173.56)	**56.26 (86.67)**^$^ **p < 0.05**		
WISCI-II score	Pre-		4.29 (5.44)	**2,92 (3.56)**^$^ **p < 0.05**	3.15 (5.58)	1.38 (2.50)^$^	1.54 (− 0.91 to 4.00)^&^	**3.43 (0.45 to 6.41)**^&^
	Post-		7.21 (5.60)		4.54 (5.71)			
	Follow-up		9.64 (4.58)	**5.35 (4.60)**^$^ **p < 0.01**	5.08 (6.07)	**1.92 (2.53)**^$^ **p < 0.05**		
SCIM-III score	Pre-		48.57 (19.50)	9.21 (9.23)* **p < 0.01**	39.23 (22.75)	9.00 (10.90)* **p < 0.05**	− 0.21 (− 7.77 to 8.20)^⌘^	− 2.20 (− 6.59 to 11.00)^⌘^
	Post-		57.79 (16.65)		48.23 (22.83)			
	Follow-up		60.93 (16.78)	12.35 (8.75)* **p < 0.001**	49.38 (23.46)	10.15 (13.17)* **p < 0.01**		

*WISCI-II* Walking Index for Spinal Cord Injury II, *SCIM III* Spinal Cord Independence Measure III, *10MWT* 10 m walk test, *TUG* Timed Up and Go test, *6MWT* 6 min’ walk test, *CI* Confidence interval). One-way repeated ANOVA and post-hoc Bonferroni (*); Friedmann test and Wilcoxon post-hoc (^$^). Intergroup comparison changes scores with t-student independent samples (^⌘^) and Mann–Whitney-U test (^&^). Bold font indicates statistical significance (*p* < 0.05)

### Motor evoked potentials

**Fig. 5:**
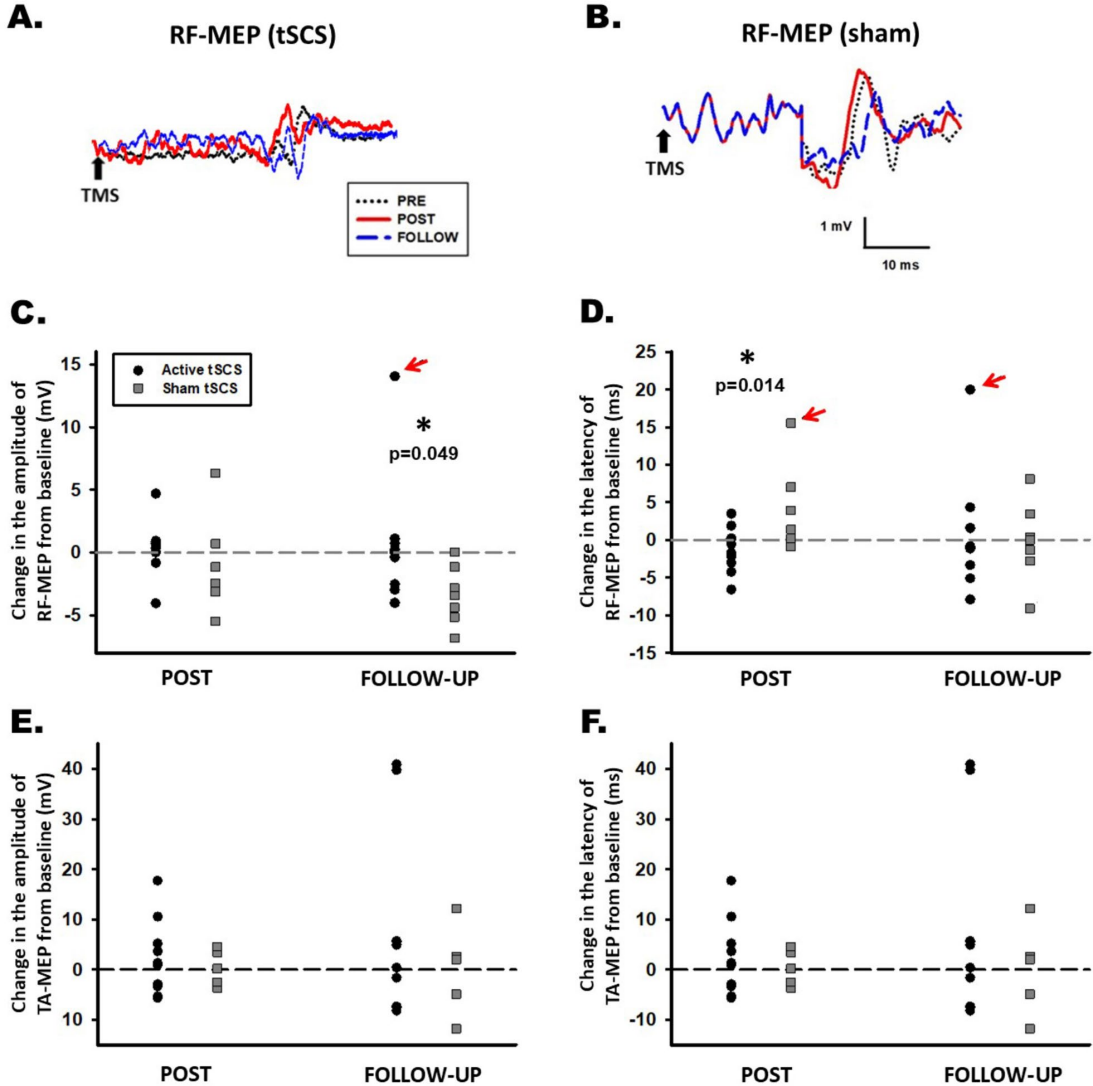
Representative motor-evoked potentials (MEP) of participant #17 (**A**: active group) and participant #23 from the sham group (**B**) recorded in the rectus femoris (RF) muscle at PRE, POST and FOLLOW. Black arrow indicates the stimulus applied by the transcranial magnetic stimulator (TMS). **C**: comparison of the effects of tSCS over amplitude of RF-MEP. **D**: comparison of the effects of tSCS over latency of RF-MEP. **E** comparison of the effects of tSCS over amplitude of tibialis anterior (TA)-MEP. **F** comparison of the effects of tSCS over latency of TA-MEP. Red arrows indicate outliers values, not included in the statistical analysis. *:p < 0.05

Motor evoked potentials (MEPs) data were missing due to lost patients at follow-up (n = 1 in the active group and n = 2 in sham group, see flow diagram), due to break-down of the TMS device (n = 2 in the active group and n = 2 in sham group) and due to the impossibility to evoke RF-MEPs (n = 2 in the active group and n = 2 in sham group) and TA-MEPs (n = 1 in the active group and n = 4 in sham group). Finally, data were obtained for RF-MEP in 9 participants in the tSCS group and in 7 participants in the sham group, while TA-MEPs were recorded 10 participants in the tSCS group and in 5 participants in the sham group. Table 4 shows the values obtained for the MEPs. There were no statistically significant differences in the mean MEP motor thresholds for the RF and TA muscles. There were no statistically significant differences in the peak-to-peak amplitude or the latency in the tSCS group. However, there was a significant decrease in the RF-MEP amplitude within the sham tSCS group compared to the baseline (Friedmann test and Wilcoxon post-hoc; p = 0.003). The intergroup comparison revealed statistically significant differences between the changes observed in the tSCS and sham groups for the latency of the RF-MEP (p = 0.026) after the intervention and for the peak-to-peak amplitude of the RF-MEP (Mann–Whitney-U test; p = 0.049) at follow-up. An example of a recorded RF-MEP and a graphical comparison of the effect of the intervention in both groups are showed in Fig. 5.

**Table 4:** Analyses of the motor evoked potentials (MEPs): threshold, peak-to-peak and latencies of quadriceps rectus femoris (RF) and tibialis anterior (TA). Confidence interval (CI)

Outcomes	Muscle	Assessment	Intervention group (tSCS + Lokomat)		Control Group (sham tSCS + Lokomat)		Comparison of changes Intervention vs. control Mean difference (95% CI)	
			Mean (SD)	Change score	Mean (SD)	Change score	Post	Baseline-Follow
Threshold (%)	RF	Pre-	53.64 (20.60)	− 3.18 (13.63)^$^	49.90 (9.93)	− 1.30 (2.16)^$^	− 1.88 (− 11.031 to 7.26)^&^	− 4.45 (− 13.39 to 4.48)^&^
		Post-	50.45 (13.23)		48.60 (9.36)			
		Follow-up	49.18 (12.27)	− 4.45 (12.34)^$^	49.90 (10.00)	0.00 (5.71)^$^		
	TA	Pre-	43.82 (9.80)	0.63 (4.80)^$^	47.22 (10.03)	− 0.44 (2.13)^$^	− 1.08 (− 2.55 to 4.71)^&^	1.45 (− 1.98 to 4.89)^&^
		Post-	44.45 (9.47)		46.78 (9.87)			
		Follow-up	44.27 (9.10)	0.45 (4.08)^$^	46.22 (10.23)	− 1.00 (3.00)^$^		
Peak-to-peak Amplitude (mV)	RF	Pre-	4.77 (2.44)	− 0.16 (1.67)*	7.17 (3.02)	− 0.90 (3.72)*	0.75 (− 2.39 to 3.90)^⌘^	**2.40 (0.00 to 4.80)**^⌘^ **p < 0.05**
		Post-	4.61 (1.38)		6.27 (3.41)			
		Follow-up	3.79 (0.85)	− 0.97 (1.82)*	3.78 (1.49)	− 3.39 (2.34)* ***p* < 0.01**		
	TA	Pre-	14.76 (7.81)	2.22 (7.45)^$^	14.69 (16.39)	0.40 (3.60)^$^	1.82 (− 9.76 to 6.12)^&^	− 7.26 (− 26.76 to 12.24)^&^
		Post-	16.98 (8.29)		15.09 (16.18)			
		Follow-up	22.03 (22.72)	7.27 (18.26)^$^	14.70 (10.78)	0.01 (8.97)^$^		
Latency (ms)	RF	Pre-	33.38 (5.31)	− 1.31 (3.29)*	32.43 (3.97)	2.59 (2.92)*	− **3.92 (**− **7.66 to **− **0.17)**^⌘^ **p < 0.05**	− 1.95 (− 7.49 to 3.58)^⌘^
		Post-	32.12 (4.67)		35.02 (4.40)			
		Follow-up	31.71 (4.39)	− 1.67(3.80)*	32.44 (5.23)	0.01 (5.80)*		
	TA	Pre-	46.43 (7.36)	− 3.08 (4.20)*	46.68 (13.87)	7.45 (30.45)*	− 10.53 (− 31.10 to 9.50)^⌘^	1.40 (− 6.62 to 9.42)^⌘^
		Post-	43.36 (6.39)		54,13 (18.41)			
		Follow-up	43.76 (5.29)	− 2.67 (3.93)*	42.48 (7.82)	− 4.20 (10.66)*		

One-way repeated ANOVA and post-hoc Bonferroni (*); Friedmann test and Wilcoxon post-hoc (^$^). Intergroup comparison changes scores with t-student independent samples (^⌘^) and Mann–Whitney-U test (^&^). Bold font indicates statistical significance (*p* < 0.05)

### Success of blinding

The overall analysis with James’ index revealed successful blinding for both participants and assessor. However, according to the calculated Bang’s index, participants in the tSCS group were not adequately blinded, and participants in the sham tSCS group showed an opposite guess, which means that they believe that they were also in the stimulation group. On the other hand, the blinding of the assessor was successful for the interventions in the tSCS group, but she was not correctly blinded when sham tSCS was applied. Data and analyses of the success of blinding are reported in Tables 5 and 6, respectively.

**Table 5:** Blinding assessment. Absolutes values and percentages of answers selected by participants and assessor

Subjects’ guess, n (%)						
Intervention	Strongly active tSCS	Somewhat active tSCS	Strongly sham tSCS	Somewhat sham tSCS	Do not know	Total
Active tSCS	11 (40.7%)	1 (3.7%)	0 (0%)	1 (3.7%)	1 (3.7%)	14 (51.85%)
Sham tSCS	9 (33.3%)	0 (0%)	0 (0%)	1 (3.7%)	3 (11.11%)	13 (48.14%)
Total	20 (74.07%)	1 (3.7%)	0 (0%)	2 (7.4%)	4 (14.8%)	27 (100%)
Assessor’s guess, n (%)						
Intervention	Strongly actived tSCS	Somewhat active tSCS	Strongly sham tSCS	Somewhat sham tSCS	Do not know	Total
Active tSCS	0 (0%)	1 (3.7%)	0 (0%)	2 (7.4%)	11 (40.7%)	14
Sham tSCS	0 (0%)	0 (0%)	0 (0%)	4 (14.8%)	9 (33.3%)	13
Total	0 (0%)	1 (3.7%)	0 (0%)	6 (22.22%)	20 (74.07%)	27

**Table 6:** Blinding assessment of participants and evaluator with James’ Blinding Index and Bang’s Blinding Index

Method	Index	*p-*value	95% Confidence Interval	Conclusion
Participants blinding				
James’ BI	0.56	0.829	(0.45, 0.67)	Blinded
Bang’s BI-Active/2 × 5	0.75	< 0.001	(0.50, 0.99)	Not blinded
Bang’s BI-Sham/2 × 5	− 0.61	1	(− 0.90, − 0.33)	Opposite guess
Assessor blinding				
James’ BI	0.83	1	(0.70, 0.93)	Blinded
Bang’s BI-Active/2 × 5	− 0.10	0.85	(− 0.27, 0.62)	Blinded
Bang’s BI-Sham/2 × 5	0.31	< 0.001	(0.09, 0.51)	Not blinded

### Adverse effects

All the participants completed the intervention and tolerated the tSCS well. The participants who underwent tSCS intervention reported mild undesired effects (85.7%; n = 12). Of those, the most reported symptoms were transitory skin redness after tSCS (100%; n = 14), lower limb paraesthesia and tingling during tSCS (28.6%; n = 4), and needles or pricking sensation under the electrodes during tSCS (57.1%; n = 8). In addition, some participants (21.4%; n = 3) reported mild adverse events related to the Lokomat intervention, such as discomfort and skin lesions in the lower limbs related to the straps.

## Discussion

This is the first controlled clinical trial to evidence a greater effect of tSCS on lower limb motor score and walking ability in individuals with iSCI when combined with RAGT, compared to sham stimulation. The combination of tSCS with Lokomat had effects on LEMS, 10MWT, TUG and WISCI-II scores 1 month after the end of the intervention. tSCS was well tolerated by participants and had no severe adverse effects. These results are in line with previous case-series and uncontrolled studies [10] that supported the feasibility and potential benefits of tSCS.

This is also the first study performed in a clinical context to assess the blinding of participants and assessors. Considering the observed results, we can argue that the protocol used in this study is valid for blinding the participants and assessor when an overall success of blinding was analyzed by the James’s Index. However, when the successful blinding of each study arm was analyzed by the Bang’s Index, this protocol did not achieve a correct blinding for the participants of the active group and the assessor of the sham group. Comparing these results with those of a previous study in non-injured volunteers [36], overall blinding improved, but the specific blinding of each study arm yielded similar results. Future studies should focus on designing intervention protocols with better blindings. The blinding of the assessor could be compromised when the recovery of one group is much better than others. To avoid this, the recruitment of an external assessor, not belonging to the hospital´s clinical staff and not familiarized with the rehabilitation procedures and patients, could be a possible solution.

The positive results observed in this study on motor score (LEMS) and gait (10MWT, TUG and WISCI-II) are in line with previous studies reporting increased lower limb motor responses [14, 15, 19, 23], greater hip flexion during the balance phase, a reduction in the amount of manual assistance during gait [15], increased TUG and WISCI-II [23], and a clinically relevant improvement in the 10MWT [20, 23, 24]. The relationship between LEMS score and gait ability has been previously established [45], and LEMS score has been proposed as a predictor of walking recovery [46, 47]. This can explain why more subjects in the tSCS group were able to walk 10 m at the end of the study and why better gait function results were observed. Previous studies have also shown a clinically relevant effect on 6MWT [23] and 2MWT [20], but in our study, the effect was not greater than that observed in the sham group for this outcome. Future controlled studies should address the impact of tSCS on endurance and aerobic capacity.

The differences in LEMS score without detected changes in dynamometry could be explained by the high variability of the recorded force of the participants. In the case of the dorsiflexor muscles, 3 participants in the tSCS group and 4 participants in the sham group were unable to generate any detectable force during the dynamometry assessment, while other participants achieved good strength scores. The position of the assessment of the quadriceps muscles could also determinate the differences. While dynamometry was assessed in the sitting position, LEMS score was assessed in supine (at the end of the range of motion). Another possible explanation could be a greater recovery of lower limb muscles not assessed by dynamometry, such as hip flexors or triceps surae muscles. Future studies should measure dynamometry of all key muscles.

Compared with those in the sham group, the observed effects were found at the 1-month follow-up, but not at the post-intervention. This fact could be of interest for understanding the mechanisms of action of tSCS. Previous studies have reported after repeated tSCS increased spinal motor evoked potentials (SMEPs), reduced motor threshold of lower limb muscles which suggest enhanced neural responsiveness over time and improvement in reflex modulation such as soleus H-reflex excitability and increased homosynaptic depression [29]. However, the duration and the implication of these mechanisms when the stimulation sessions finish are still unknown. In the clinical context, previous studies of cervical tSCS have reported that functional gains persist for more than 3 months [48, 49] but no follow-ups have been reported in clinical studies of lumbar tSCS. Studies adding tSCS to gait training programs have used 6 sessions (2 weeks) [20], 15 sessions (3 weeks) [21], 23 sessions (8 weeks) [23] and 72 sessions [16]. Although all of these studies reported positive results, only Estes et al. [20] compared to a sham stimulation group and found no between-group differences after 2 weeks (3 sessions/week) of treatment. It is possible that 6 sessions are not enough to achieve a functional effect. The optimization of the dose‒response effect needs to be addressed.

The evidence about the effectiveness of tSCS for spasticity is heterogeneous and limited [25]. Although some studies have shown a decrease in spasticity [24, 50, 51], other studies have found no effects [20] or a slight increase [18]. This study revealed no effects on hypertonia in the tSCS nor in the sham group. This response might be related to time since SCI. While the current study and the study of Estes et al. [20] recruited sub-acute participants (from 2 to 6 months after the injury, when the hypotonic phase changes to the spastic phase) [52] and observed no effects; other studies with positive effects on spasticity have recruited chronic participants (> 1 year) [18, 24, 49, 53, 54] when spasticity is well established. Another relevant point in this study is the exclusion of participants with a high level of hypertonia or spasms due to the exclusion criteria suggested for the use of Lokomat. Finally, it should be noted that most of the studies measured spasticity using the MAS scale [18, 49, 53, 54]. However, spasticity is a multidimensional phenomenon, and the MAS score measures only resistance to passive stretch (hypertonia). The inclusion of an appropriate and comprehensive battery of tests that better represent the spastic state of the participants is recommended in future studies [55].

Regarding neurophysiological outcomes, although between-group differences in the change in the amplitude of RF-MEP detected at 1-month follow-up match with the observed differences in strength and gait at the same time, amplitude of RF-MEP did not increase in the active group and the difference is due to a decrease in the sham group. This result is not in line with the increase of RF-MEP found in our previous study performed in non-injured subjects [36]. However, in individuals with SCI, Benavides et al. [56] showed no effect after one session of cervical tSCS in the biceps brachii MEP. The decrease of the amplitude in RF-MEP has not been previously reported [57–59] and a good correlation between lower limb MEPs, motor scores and ambulatory capacity has been shown during the sub-acute phase of iSCI [57].

It is important to consider that this study has been conducted in the sub-acute phase of SCI. However, most of the previous studies recruited subjects at the chronic stage [10, 26]. The study of the efficacy of tSCS during the sub-acute phase is a challenge due to the substantial number of clinical changes that occur during this phase. The study of Estes et al. [20] is the only previous one that applied tSCS at the lumbosacral level in sub-acute patients (n = 8), and the only previous one that compared to a sham group (n = 8). While they applied 6 sessions of 30 min stimulation at 50 Hz, our study applied 20 sessions of 20 min at 30 Hz. They found certain clinical effects, but they did not find statistical significance when compared to the sham group [20]. The optimization of the therapeutic window of the tSCS needs to be addressed in future studies.

Despite the significant results observed in this study, the clinical applicability of tSCS is mainly based on its integration into clinical practice and the clinical magnitude of the observed effects. The stimulators used in some previous studies used a 10 kHz carrier frequency, which is not usually available in clinical devices [17, 18, 48]. In the present study, a 30 Hz rectangular biphasic symmetric current, which is easily available by clinical devices, was used, and the combination with Lokomat was performed in a clinical setting, with good experience reported by physiotherapists and participants. Regarding clinical significance, previous studies have shown relevant clinical differences in gait function [20, 23], but they did not measure [23] or did not find [20] differences compared to sham stimulation. In our study, the minimal clinically important difference (MCID) of 0.13 m/s [60] was exceeded for the 10MWT (estimated in our study at 0.26 m/s); for the TUG test, the MCID of 10.8 s [60] was also reached (47.7 s). For the WISCI-II, a 2-point increase has been proposed as clinically significant [61] while the observed change compared to the sham group in our study was 3.4 points. Regarding the LEMS score, the 3.4-point difference found in this study almost reached the MCID of 3.6 identified for this measure [62]. Furthermore, the difference observed here aligns with the differences from the control or sham groups observed in other clinically implemented adjunctive therapies for iSCI individuals, such as robotic gait training (3.1 points) [61] functional electrical stimulation (4.6 points) [63] or transcranial magnetic stimulation (2.3 points) [9].

### Limitations

The primary limitation of this randomized clinical trial is its small sample size (n = 27), which could underestimate potentials effects that have not reached statistical significance, possibly due to a high type-2 error. This limitation is attributed to challenges in recruiting subjects within the designated recruitment period, which extended for over 2 years. However, it must be noted that all previous studies recruited a small sample size (n < 10) [10, 25] without including a control group. The difficulties and the low recruitment rate (17%) have been highlighted in a previous controlled study.^11^ Although we are aware that current clinical trials are undergoing with targeting larger sample size [64] our study has achieved a 25% of recruitment rate. To our knowledge, this is the largest sample size reported to date in a controlled study of tSCS. On the other hand, probably due to the low sample size, our cohort of participants showed differences at baseline in % body weight support and sex, and a higher proportion of AIS D participants in the active group compared to the sham group. Future work should aim to minimize these differences to reduce potential confounding effects.

The stimulation intensity of the current applied during active tSCS was not objectively determined. We chose to use the tolerance threshold to ensure safety, to maintain patient adherence to the treatment and to have a better translation to the clinical setting. However, subjective methods based on the subject’s sensation have been criticized [65]. The posterior root-muscle reflex has been previously used to determine the intensity of tSCS [36] but this method is not suitable for clinical practice. Although Kumru et al. [66] highlighted the importance of intensity in a cervical tSCS protocol for non-injured subjects, the optimization of the intensity parameter has not been studied in clinical research.

The outcomes associated with MEPs were not analysed in an intention-to-treat way because MEPs could not be induced in some patients at baseline, and there was trouble with a breakdown of the TMS device. For these reasons, several data of MEPs were lost, and the sample size of this outcome is very small. The results of MEPs should be interpreted with caution. On the other hand, although the intention to treat analysis performed for clinical measures is usually recommended in clinical studies, the imputed data is a challenge due to the high number of patients who were unable to complete gait tests at the baseline. The choice of assigning the worst value of all subjects for participants unable to complete the test (for the 10MWT and TUG) could overestimate the magnitude of the effect and the type-1 error of these outcomes. The magnitude of the differences observed in gait tests should be interpreted with caution.

## Conclusions

The application of 20 sessions of tSCS over T11-T12 vertebrae combined with RAGT showed a greater improvement in lower limb motor score and gait recovery in subacute iSCI subjects at 1 month follow-up than the application of sham stimulation, without affecting hypertonia. Furthermore, the sham stimulation protocol used in this study was valid for blinding of participants. tSCS is a non-invasive and safe therapy without severe adverse effects that can be easily applied in conjunction with other locomotor strategies in a clinical setting. However, further studies are needed to investigate the optimal dose, parameters, and intensity of the current in larger populations.

## Supplementary Information

Supplementary Material 1.

## Data Availability

The data generated in this study have been deposited in the Zenodo database under accession code 10.5281/zenodo.10995944.

## Abbreviations

tSCS: Transcutaneous spinal cord stimulation
RAGT: Robotic-assisted gait training
iSCI: Incomplete spinal cord injury
LEMS: Lower extremity motor score
10MWT: 10-Meter Walk tests
TUG: Timed Up and Go test
6MWT: 6-Minute Walk test
SCIM III: Spinal Cord Independence Measure III
WISCI-II: Walking Index for Spinal Cord Injury II
AIS: American Spinal Injury Association Impairment Scale
MVC: Maximal voluntary contraction
MAS: Modified Ashworth Scale
MEP: Motor evoked potentials
RF: Rectus femoris
TA: Tibialis anterior
TMS: Transcranial magnetic stimulation
MCID: Minimal clinically important difference
