# Supplementary material for: Transcutaneous spinal cord stimulation combined with robotic-assisted body weight-supported treadmill training enhances motor score and gait recovery in incomplete spinal cord injury: a double-blind randomized controlled clinical trial
Source: J Neuroeng Rehabil. 2025 Jan 30;22:15. doi: 10.1186/s12984-025-01545-8 (PMC11780808; doi:10.1186/s12984-025-01545-8)
Supplement: Supplementary file 1 — Supplementary Material 1. [file 12984_2025_1545_MOESM1_ESM.docx]

**SUPPLEMENTARY MATERIAL 1**

Sociodemographic and clinical characteristics of each SCI patient.

| **Subject** | **Intervention** | **Age (y)** | **Gender** | **Weight**  **(kg)** | **Height**  **(cm)** | **SCI**  **Level** | **AIS** | **%**  **BWS**  **1^st^ ss** | **%**  **BWS**  **20^th^ ss** | **Time since SCI (days)** | **SCI**  **Aetiology** |
| --- | --- | --- | --- | --- | --- | --- | --- | --- | --- | --- | --- |
| 1 | tscs +  Lokomat | 57 | M | 65 | 1,65 | T9 | D | 40 | 25 | 56 | Post-surgery |
| 2 | tSCS +  Lokomat | 21 | M | 73 | 1,8 | C4 | D | 40 | 35 | 165 | Inflammatory |
| 3 | tSCS +  Lokomat | 43 | M | 75 | 1,65 | T10 | D | 40 | 20 | 85 | Vascular |
| 4 | tSCS +  Lokomat | 44 | M | 117 | 1,86 | T4 | C | 60 | 50 | 139 | Trauma |
| 7 | tSCS +  Lokomat | 61 | M | 90 | 1,79 | T10 | C | 45 | 35 | 146 | Inflammatory |
| 8 | tSCS +  Lokomat | 36 | M | 70 | 1,74 | C6 | C | 35 | 30 | 90 | Trauma |
| 9 | tSCS +  Lokomat | 53 | M | 98 | 1,75 | C4 | D | 50 | 45 | 103 | Trauma |
| 12 | tSCS +  Lokomat | 53 | M | 75 | 1,7 | C4 | D | 30 | 20 | 59 | Trauma |
| 13 | tSCS +  Lokomat | 39 | M | 80 | 1,82 | C4 | C | 50 | 35 | 110 | Trauma |
| 18 | tSCS +  Lokomat | 71 | M | 97 | 1,8 | T10 | C | 45 | 40 | 123 | Post-surgery |
| 19 | tSCS +  Lokomat | 62 | M | 100 | 1,78 | C4 | C | 50 | 50 | 120 | Trauma |
| 20 | tSCS +  Lokomat | 29 | M | 75 | 1,72 | T10 | C | 35 | 25 | 127 | Vascular |
| 22 | tSCS +  Lokomat | 34 | M | 67 | 1,87 | C4 | D | 25 | 20 | 146 | Trauma |
| 24 | tSCS +  Lokomat | 60 | M | 75 | 1,67 | T6 | C | 35 | 25 | 59 | Inflammatory |
| 21 | Sham tSCS  +Lokomat | 40 | M | 90 | 1,98 | C4 | C | 40 | 30 | 60 | Trauma |
| 23 | Sham tSCS +  Lokomat | 57 | M | 105 | 1,78 | C4 | C | 50 | 30 | 121 | Trauma |
| 25 | Sham tSCS +  Lokomat | 59 | F | 52 | 1,57 | T11 | C | 25 | 25 | 159 | Vascular |
| 16 | Sham tSCS +  Lokomat | 34 | M | 65 | 1,7 | C4 | C | 30 | 20 | 98 | Trauma |
| 26 | Sham tSCS +  Lokomat | 76 | F | 45 | 1,5 | C5 | D | 25 | 25 | 39 | Post-surgery |
| 17 | Sham tSCS +  Lokomat | 36 | M | 70 | 1,78 | C4 | C | 35 | 35 | 66 | Trauma |
| 27 | Sham tSCS +  Lokomat | 27 | M | 75 | 1,7 | C8 | D | 35 | 25 | 140 | Vascular |
| 5 | Sham tSCS +  Lokomat | 61 | M | 90 | 1,75 | T10 | C | 45 | 35 | 160 | Vascular |
| 6 | Sham tSCS +  Lokomat | 49 | M | 78 | 1,78 | T8 | D | 30 | 25 | 64 | Vascular |
| 10 | Sham tSCS +  Lokomat | 38 | M | 67 | 1,85 | C4 | C | 35 | 25 | 88 | Trauma |
| 11 | Sham tSCS +  Lokomat | 23 | M | 62 | 1,8 | C5 | C | 35 | 25 | 177 | Trauma |
| 14 | Sham tSCS +  Lokomat | 67 | M | 54 | 1,6 | C2 | C | 30 | 25 | 145 | Trauma |
| 15 | Sham tSCS +  Lokomat | 66 | F | 60 | 1,57 | C4 | C | 30 | 30 | 161 | Trauma |
| ***p-value*** | | 0.82**^a^** | 0.057**^b^** | 0.057**^a^** | 0.37**^a^** | 0.365**^b^** | 0.276**^b^** | 0.035^a^ | 0.063**^a^** | 0.777**^a^** | 0.258^b^ |

**Note:** AAmerican Spinal Cord Injury Association Impairment Scale (AIS); Male (M); Female (F); % Body weight support (BWS). Statistical test: a) Student’s t-test independent samples. b) Pearson's chi-squared test.
